# Comparison of HIV-1 Genotypic Resistance Test Interpretation Systems in Predicting Virological Outcomes Over Time

**DOI:** 10.1371/journal.pone.0011505

**Published:** 2010-07-09

**Authors:** Dineke Frentz, Charles A. B. Boucher, Matthias Assel, Andrea De Luca, Massimiliano Fabbiani, Francesca Incardona, Pieter Libin, Nino Manca, Viktor Müller, Breanndán Ó. Nualláin, Roger Paredes, Mattia Prosperi, Eugenia Quiros-Roldan, Lidia Ruiz, Peter M. A. Sloot, Carlo Torti, Anne-Mieke Vandamme, Kristel Van Laethem, Maurizio Zazzi, David A. M. C. van de Vijver

**Affiliations:** 1 Virology, Erasmus MC, University Medical Centre Rotterdam, Rotterdam, The Netherlands; 2 Medical Microbiology, University Medical Centre Utrecht, Utrecht, The Netherlands; 3 High Performance Computing Center Stuttgart, University of Stuttgart, Stuttgart, Germany; 4 Catholic University of the Sacred Heart, Institute of Clinical Infectious Diseases, Rome, Italy; 5 Infectious Diseases Unit 2, University Hospital of Siena, Siena, Italy; 6 Informa SrL, Rome, Italy; 7 MyBioData, Rotselaar, Belgium; 8 Institute of Microbiology, School of Medicine, University of Brescia, Brescia, Italy; 9 Institute of Biology, Eötvös Loránd University, Budapest, Hungary; 10 Computational Science, University of Amsterdam, Amsterdam, The Netherlands; 11 Institut de Recerca de la SIDA irsiCaixa, Badalona, Spain; 12 Clinic of Infectious Diseases Catholic University, Rome, Italy; 13 Institute of Infectious and Tropical Diseases, School of Medicine, University of Brescia, Brescia, Italy; 14 Department of Molecular Biology, University of Siena, Siena, Italy; 15 Rega Institute, Katholieke Universiteit Leuven, Leuven, Belgium; Singapore Immunology Network, Singapore

## Abstract

**Background:**

Several decision support systems have been developed to interpret HIV-1 drug resistance genotyping results. This study compares the ability of the most commonly used systems (ANRS, Rega, and Stanford's HIVdb) to predict virological outcome at 12, 24, and 48 weeks.

**Methodology/Principal Findings:**

Included were 3763 treatment-change episodes (TCEs) for which a HIV-1 genotype was available at the time of changing treatment with at least one follow-up viral load measurement. Genotypic susceptibility scores for the active regimens were calculated using scores defined by each interpretation system. Using logistic regression, we determined the association between the genotypic susceptibility score and proportion of TCEs having an undetectable viral load (<50 copies/ml) at 12 (8–16) weeks (2152 TCEs), 24 (16–32) weeks (2570 TCEs), and 48 (44–52) weeks (1083 TCEs). The Area under the ROC curve was calculated using a 10-fold cross-validation to compare the different interpretation systems regarding the sensitivity and specificity for predicting undetectable viral load. The mean genotypic susceptibility score of the systems was slightly smaller for HIVdb, with 1.92±1.17, compared to Rega and ANRS, with 2.22±1.09 and 2.23±1.05, respectively. However, similar odds ratio's were found for the association between each-unit increase in genotypic susceptibility score and undetectable viral load at week 12; 1.6 [95% confidence interval 1.5–1.7] for HIVdb, 1.7 [1.5–1.8] for ANRS, and 1.7 [1.9–1.6] for Rega. Odds ratio's increased over time, but remained comparable (odds ratio's ranging between 1.9–2.1 at 24 weeks and 1.9–2.2 at 48 weeks). The Area under the curve of the ROC did not differ between the systems at all time points; p = 0.60 at week 12, p = 0.71 at week 24, and p = 0.97 at week 48.

**Conclusions/Significance:**

Three commonly used HIV drug resistance interpretation systems ANRS, Rega and HIVdb predict virological response at 12, 24, and 48 weeks, after change of treatment to the same extent.

## Introduction

The effectiveness of antiretroviral therapy has been limited by the development of HIV-1 drug resistance. Resistance occurs frequently in patients and may decrease both the magnitude and the duration of the response to treatment [1].

Several prospective studies have shown that the use of genotypic resistance analysis to guide the new treatment choice for patients failing their current HAART improves virologic outcome [2], [3], [4], [5]. The complex mutational patterns are however difficult to interpret, due to the many different drug resistance mutations [6] and the varying levels of decreased susceptibility of these mutations to different drugs. This led to the development of several interpretation systems [7], which provide rules to help physicians interpret HIV-1 drug resistance genotyping results.

ANRS, Stanford HIVdb, and Rega are the three most commonly used and publicly available drug resistance interpretation systems, which are all regularly updated. The systems are rule based algorithms, providing scores for specific (combinations of) mutations. The scores are then translated into different levels of susceptibility. The rules for these scores are based on literature and expert's opinion. The Rega system was the first to be validated in drug experienced patients [8], [9], followed by ANRS [5], [9] and Stanford [9].

A good way to compare systems is by using virological response data in correlation with the prediction of interpretation systems. However, some systems may be better for short-term virological outcomes, and others may be better for longer-term outcomes. The results of a comparison between systems may therefore depend on the virological outcome time point that is used. In this study, a large data set of HIV-1 patient's sequences was collected together with virological data to compare the three most commonly used interpretation systems in genotypic susceptibility score and in the prediction of virological response. We used 3 different virological outcome time points to analyze the effect of therapy duration on the prediction of systems.

## Methods

### Study population

Data was made available through the EU-sponsored ViroLab and EuResist projects [10], [11], [12]. The ViroLab project comprises data from Belgium (Katholieke Universiteit Leuven), Italy (University of Brescia and Catholic University of the Sacred Heart of Roma), Spain (IrsiCaixa Badalona), and The Netherlands (Erasmus Medical Centre Rotterdam). The EuResist project consists of data from Italy (ARCA database; http://www.hivarca.net/), Germany (AREVIR database); Sweden (Karolinska Infectious Diseases and Clinical Virology Department), and Luxembourg (Retrovirology Laboratory, CRP-Santé). The time-periods of available therapies in the ViroLab and EuResist database ranged between 1996 and 2008.

**Figure 1:**
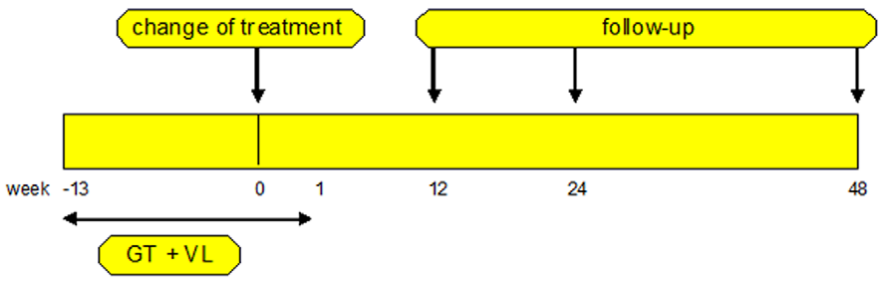
Schematic definition of a treatment change episode. The treatment change episode requirements are as follows: (1) a baseline genotypic drug-resistance and viral load test between 90 days before and 8 days after change of therapy (2) at least one follow-up viral load measurement at 12 (8–16), 24 (16–32), or 48 (44–52) weeks.

These databases were used to extract treatment change episodes (TCEs). TCEs were defined, in patients aged ≥18, as follows (figure 1): (1) a baseline genotype (Reverse transcriptase and Protease region) and viral load (detectable being >50 copies/ml) obtained within 90 days before and 8 days after treatment change; (2) at least one follow-up viral load measurement at 12 (range: 8–16), 24 (16–32), or 48 (44–52) weeks; (3) no changes in therapy between the time of the baseline viral load and the follow-up viral load measurement. In case more genotypic tests or viral load measurements were performed within an analyzed treatment period, the value closest to the start of therapy or the follow-up measurement time was used.

### Interpretation systems and genotypic susceptibility scores (GSSs)

The genotypic results were interpreted using three commonly used rule-base interpretation systems: Agence Nationale de recherches sur le SIDA (ANRS) version 17; Stanford HIVdb, version 5.1.2; and Rega Institute version 8.0.1. The ANRS and Rega both report 3 levels of resistance: susceptible, intermediate, and resistant. For ANRS, we translated the definitions ‘susceptible’, ‘intermediate’, and ‘resistant’ into susceptibility scores of 1, 0.5, and 0, respectively. For the Rega scores, we used the weighted score suggested by Rega, which uses the following changes: NNRTI were scored 0.25 (with the exception of etravirine with a score of 0.5) for intermediate resistance, and ritonavir-boosted PI were scored 0.75 and 1.5 for intermediate resistance and susceptible, respectively. The Stanford algorithm uses 5 levels of resistance. We assigned the following scores to these 5 levels of Stanford: 0, 0.25, 0.50, 0.75, and 1 for respectively the high-level resistance, intermediate resistance, low-level resistance, potential low-level resistance, and susceptible. In a separate analysis we used the unweighted scores for Rega. We assigned the scores 0, 0.5, and 1 to the ‘resistant’, ‘intermediate’, and ‘susceptible’ groups for all drugs, respectively. The three systems did not include a score for ritonavir. We therefore excluded eleven TCEs that used ritonavir as only protease inhibitor, as we could not calculate a GSS of their treatment regimens.

The arithmetic sum of the individual score for the specific drugs provided the total GSS of that treatment. For brevity, we classified the total GSS score in the following categories: 0 to <1, 1 to <2, 2 to <3, 3 to <4, and ≥4. The 0 to <1 group contains viral sequences almost entirely resistant to the drugs in their regimen, and the ≥4 group contains viral sequences susceptible to more than 3 drugs given in their regimen.

To calculate the prevalence of drug resistance we used the mutation list published by the International AIDS Society USA (IAS-USA) [13].

### Statistical analysis

Kaplan-Meier curves were estimated to determine the association between GSSs and the proportion of TCEs having an undetectable viral load (<50 copies/ml). The association between GSS scores and undetectable viral load was analyzed with a logistic regression. In the multivariate analyses we adjusted for real time to viral load measurement (i.e. number of days between the TCEs and the follow-up viral load measurement) and log viral load at start of therapy. Furthermore, we used logistic regression, to calculate Odds Ratios for each GSS group compared to the GSS group of 0 to <1. The receiver operating characteristic (ROC) curves were calculated to analyze the trade-off between the proportion of true-positive (correct virologic response prediction) and false-positive (incorrect virologic response prediction) results across the range of possible prediction cutoffs. The AUC (Area Under the Curve) is a value between 0 and 1 that corresponds to the probability that a randomly selected virologic success receives a higher score than a randomly selected virologic failure. We used the AUCs to calculate how well the systems separate the GSS groups into those with and without undetectable viral load (<50 copies/ml). Robust extra-sample error estimation was obtained by 10-fold cross-validation [14]. We compared the multiple independent runs of the 10-fold cross validation results with a Kruskal-Wallis test. Analyses were performed with the SPSS software package (version 15.0 for Windows, SPSS).

## Results

### Baseline characteristics of the study population

The baseline characteristics are shown in Table 1. We included 3131 patients in our study, of which most were male (73%), most were infected with subtype B viruses (81.9%), and the median age was 39 years (range 18–78). Of the 3131 patients, 476 (12.7%) had more than one TCE, which leads to a total of 3,763 TCEs included in the study. Of these TCEs, 2,152 had a viral load measurement at week 12, 2,570 at week 24, and 1,083 at week 48. TCEs were retrospectively included between 1996 and 2008. Most TCEs (2085, 55.4%) were included between 2001 and 2004, and fewer TCEs were included between 2005 and 2008 (1029, 27.3%) and between 1996 and 2000 (649, 17.2%). The median HIV RNA level of the TCEs was 4.43 log_10_ copies/ml [interquartile range (IQR), 3.65–5.08], and the median CD4^+^ cell count was 233 cells/µL (IQR, 120–371 cells/µL). The most commonly given treatments were lamivudine (59%), tenofovir (37%), and lopinavir (35%). A combination of lamivudine, zidovudine, and lopinavir/r was the most frequently given therapy combination, with a percentage of 8%, followed by 6% for the therapy combination lamivudine, tenofovir, and lopinavir/r.

**Table 1:** Baseline patient characteristics.

**Number of patients**		3131
**Male,** number (%)		272 (72.6)
**Age,** median (IQR^*^)		39 (18–78)
**HIV-1 subtype,** number (%)	Subtype B	2563 (81.9)
	Subtype A	158 (5.0)
	Subtype G	118 (3.8)
	Subtype C	90 (2.9)
	other	76 (2.4)
	Subtype F	62 (1.9)
	CRF 02_AG	28 (0.9)
	CRF 12_BF	24 (0.8)
	unclassified	12 (0.4)
**Number of treatment-change episodes**		3763
**Baseline CD4 count** (cells/mm^3^), median (IQR)		233 (120–371)
**Baseline viral load** (log_10_)(copies/ml), median (IQR)		4.43 (3.65–5.08)
		Number (%)
**Treatment-change episodes**	1 treatment-change episode	1555 (41.3)
	>1 treatment-change episodes	476 (12.6)
	>2 treatment-change episodes	108 (2.9)
**Year of treatment**	1996–2000	649 (17.2)
	2001–2004	2085 (55.4)
	2005–2008	1029 (27.3)
**NRTI Drug treatment**	lamivudine	2224 (59)
	tenofovir	1400 (37)
	zidovudine	1082 (29)
	didanosine	1007 (27)
	stavudine	932 (25)
	abacavir	590 (16)
	didanosine	653 (18)
	emtricitabine	246 (7)
**NNRTI Drug treatment**	efavirenz	660 (18)
	nevirapine	447 (12)
	etravirine	1 (0)
	delavirdine	1 (0)
**PI Drug treatment**	lopinavir	1309 (35)
	nelfinavir	332 (9)
	atazanavir	274 (7)
	indinavir	263 (7)
	saquinavir	221 (6)
	amprenavir	202 (5)
	tipranavir	70 (2)
	darunavir	28 (1)
**Other drug treatment**	T20	135 (4)
**therapy combinations**	lamivudine + lopinavir + zidovudine	315 (8)
	lamivudine + lopinavir + tenofovir	244 (6)
	lamivudine + zidovudine + abacavir	133 (4)
	lamivudine + tenofovir + efavirenz	133 (4)
	lamivudine + zidovudine + efavirenz	114 (3)
	tenofovir + lopinavir + didanosine	102 (3)

*IQR is interquartile range.

### Prevalence of mutations at baseline

**Figure 2:**
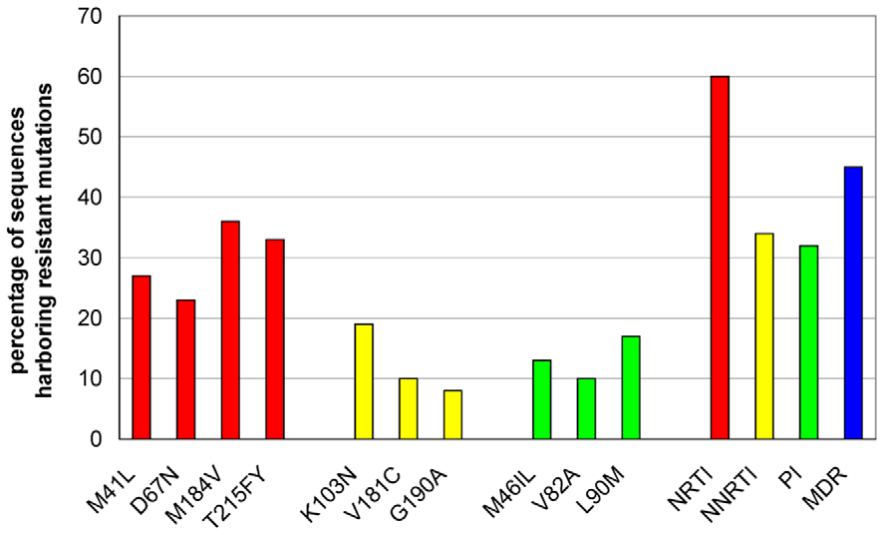
Drug resistance prevalences. Percentage of sequences having resistance mutations to NRTI (red), NNRTI (yellow), PI (green), and Multi drug resistance (MDR) (blue).

The percentage of sequences having a drug resistance mutation is shown in Figure 2. NRTI resistance associated mutations were most frequently found with a prevalence of 62% [13]. The most prevalent NRTI resistance mutations were M41L (27.0%), D67N (23.2%), M184V (35.6%), and T215FY (32.9%). Mutations associated with resistance to NNRTI and PI, were detected less frequently, in 34% and 32% of the cases, respectively. K103N (18.6%), V181C (10.2%), and G190A (8.0%) were the most prevalent NNRTI mutations. The PI mutations with highest prevalences were M46IL (13.2%), V82A (9.6%), and L90M (16.9%). The comparisons of the mutation patterns showed no substantial differences between TCEs with a follow-up viral load at 12, 24, and 48 weeks.

### Genotypic Susceptibility Score distribution

**Figure 3:**
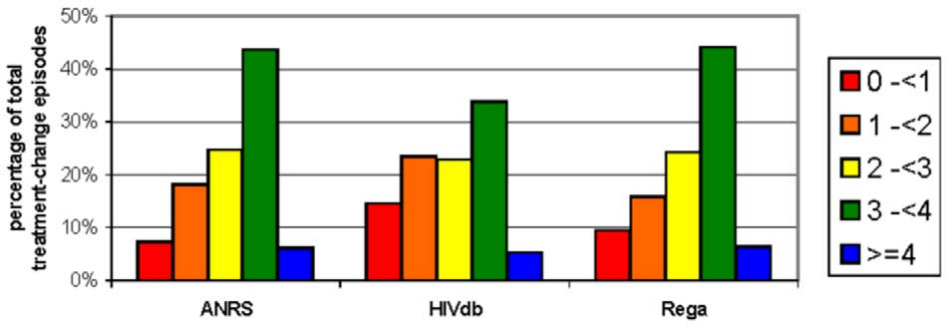
Total Genotypic Susceptibility Scores for ANRS, HIVdb, and Rega. Total Genotypic Susceptibility Scores were calculated using the arithmetic sum of the individual scores given by the systems for each specific drug given in a regimen. We classified the GSS score for ANRS, HIVdb, and Rega in the following categories: 0 to <1, 1 to <2, 2 to <3, 3 to <4, and ≥4. GSS scores were calculated for 3759 TCEs.

The genotypic susceptibility scores for a TCE was calculated as the total score of genotypic susceptibility scores for all drugs in one regimen as explained in the ‘method’ section. Figure 3 displays the proportions of cases in each susceptibility category, according to ANRS, HIVdb, and Rega. All systems show that at least three active drugs were started in a large proportion of TCEs. The mean GSS of the three systems were slightly smaller for HIVdb, with 1.92±1.17, compared to Rega and ANRS, with 2.22±1.09 and 2.23±1.05, respectively. The unweighted Rega scores did not differ much from the other scores with a mean of 2.15±1.09.

The GSS of TCEs with longer follow-up were slightly higher compared to TCEs with a short follow-up time (data not shown), with baseline GSS means ranging between 1.93 and 2.23 at 12 weeks, 1.98 and 2.29 at 24 weeks, and 1.98 and 2.32 for TCEs with viral load measurement available at 48 weeks.

### Prediction of virologic outcomes

The virologic responses of all TCEs are described in Table 2. The percentage of an undetectable viral load (<50 copies/ml) was higher in week 24 compared to week 12. Week 48 did not show a large increase in percentage compared to week 24. TCEs with higher Genotypic Susceptibility Score had a higher change of reaching an undetectable level of viral load. At 48 weeks, in more than 70% of the TCEs with a Genotypic Susceptibility Score of ≥4, the viral load became undetectable.

**Table 2:** The viral load response and GSS groups at different time points.

	ANRS			HIVdb			Rega		
week	12	24	48	12	24	48	12	24	48
GSS 0-<1	7.3	8.3	8.1	12.4	14.8	18.0	10.0	9.3	12.0
GSS 1-<2	23.7	30.0	33.5	28.6	39.4	41.6	19.7	26.4	27.4
GSS 2-<3	36.7	47.6	51.7	44.7	55.9	61.7	39.2	50.2	54.9
GSS 3-<4	46.2	64.4	66.7	47.1	66.4	68.1	46.0	65.0	67.2
GSS ≥4	47.2	69.0	74.6	45.0	68.0	72.0	47.3	65.1	72.1

The percentages of treatment-change episodes with an undetectable viral load (<50 copies/ml) are shown for each GSS group at week 12, week 24, and week 48 for ANRS, HIVdb and Rega.

**Figure 4:**
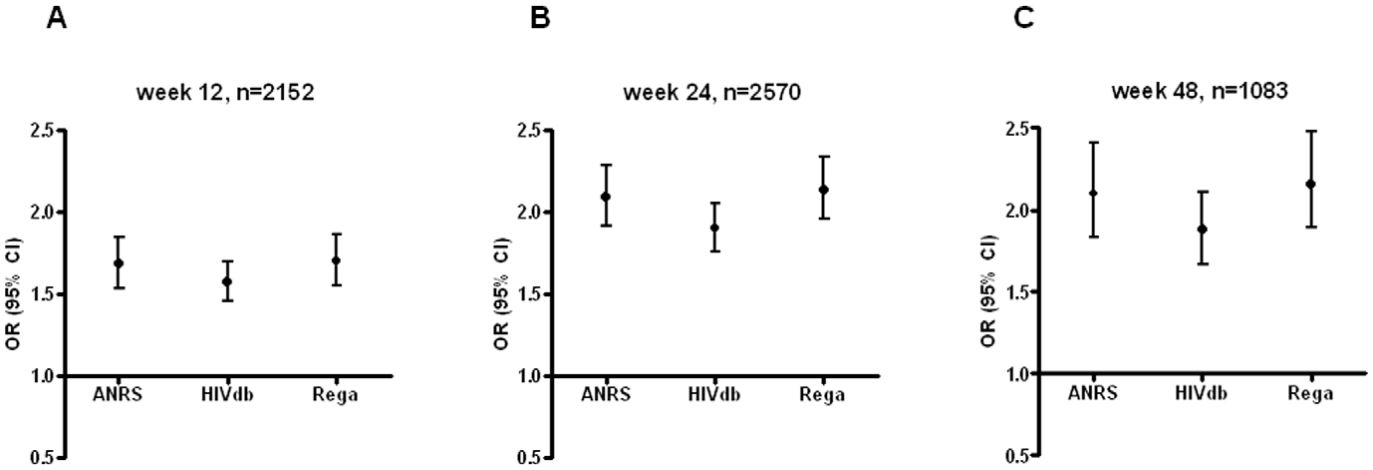
Association between Genotypic Susceptibility Score and undetectable viral load. The adjusted odds ratios (ORs) with 95% confidence intervals for RNA levels <50 copies/ml at (A) 12 weeks, (B) 24 weeks, and (C) 48 weeks per unit increase of GSS according to ANRS, HIVdb, and Rega. These odds ratios were adjusted for log viral load at start of therapy and real time to viral load measurement, and similar to the unadjusted odds ratios.

Adjusted odds ratios for reaching a viral load below 50 copies/mL for each unit increase in GSS are reported in figure 4. These predictions of the virological response were similar to the odds ratios without adjusting for log viral load at start of therapy and real time to viral load measurement (data not shown). At all time points, the interpretation systems were significantly predictive of the virological response. Odds Ratios for each unit increase of the GSSs ranged from 1.77 (95% Confidence Intervals (CI): 1.62–1.94), 1.87 (95%CI: 1.69–2.06), and 1.88 (95%CI: 1.70–2.08) at 12 weeks to around 1.99 (95% CI: 1.84–2.16), 2.20 (95%CI: 2.01–2.41), and 2.16 (95%CI: 1.97–2.37) at 24 weeks for HIVdb, Rega, and ANRS, respectively. Furthermore, the Odds Ratios for the unweighted Rega scores were similar, ranging between 1.86 (95% CI: 1.69–2.05) at week 12 and 2.16 (95% CI: 1.98–2.36) at week 24.

**Figure 5:**
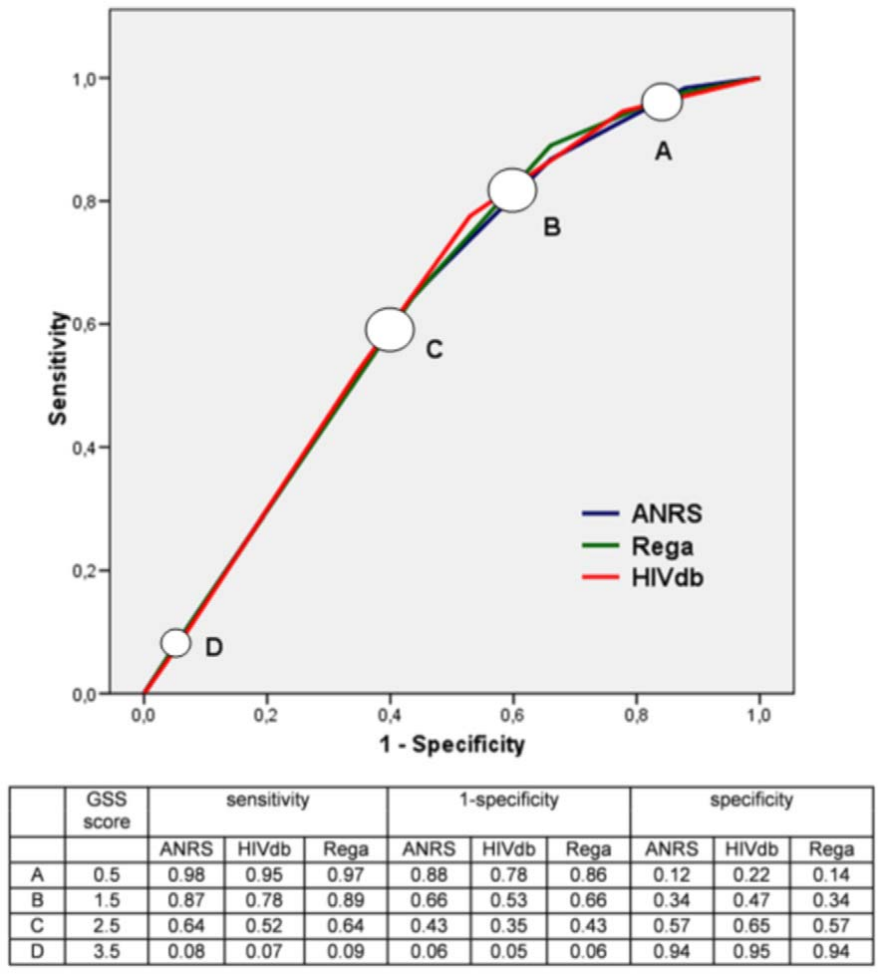
ROC curves for the logistic models for ANRS, HIVdb, and Rega at 12 weeks. The sensitivity, 1-specificity, and specificity are given in the table for the cut-off points 0.5 (A), 1.5 (B), 2.5 (C), and 3.5 (D) for ANRS, HIVdb, and Rega.

The ROC curves in figure 5 depict different cut-off points, for the three interpretation systems. In the table below the graph, the sensitivity, 1-specificity, and specificity are given for these cut-off points. The sensitivity and specificity of the ROC curves for the systems are all similar. The calculated AUCs were around 0.63 at week 12 and 0.68 at week 24 and 48 (shown in Table 3). These AUCs did not significantly differ among the systems (with p-values ranging between 0.60–0.97) at all time points. The AUCs of the unweighted Rega did not differ from the normal ANRS, HIVdb, and Rega scores, with means of 0.63 at week 12 and 0.68 at week 24 and 48. (data not shown).

**Table 3:** Multiple cross-validation for calculating AUC for the different interpretation systems.

week	system	AUC^*^	sd	Kruskal-Wallis test	
				Chi-square	p-value
Week 12	ANRS	0.629	0.05	0.280	0.597
	HIVdb	0.634	0.05		
	Rega	0.620	0.05		
Week 24	ANRS	0.677	0.04	0.143	0.705
	HIVdb	0.689	0.03		
	Rega	0.689	0.03		
Week 48	ANRS	0.671	0.06	0.001	0.970
	HIVdb	0.680	0.06		
	Rega	0.679	0.06		
All weeks	ANRS	0.671	0.03	0.322	0.570
	HIVdb	0.680	0.03		
	Rega	0.680	0.02		

*AUCs (area under the receiver operating characteristic curve) were obtained from 10-fold cross-validated predictions. AUCs of 0.5 indicate that the interpretation system is not an explanatory factor for the percentage undetectable viral load.

**Figure 6:**
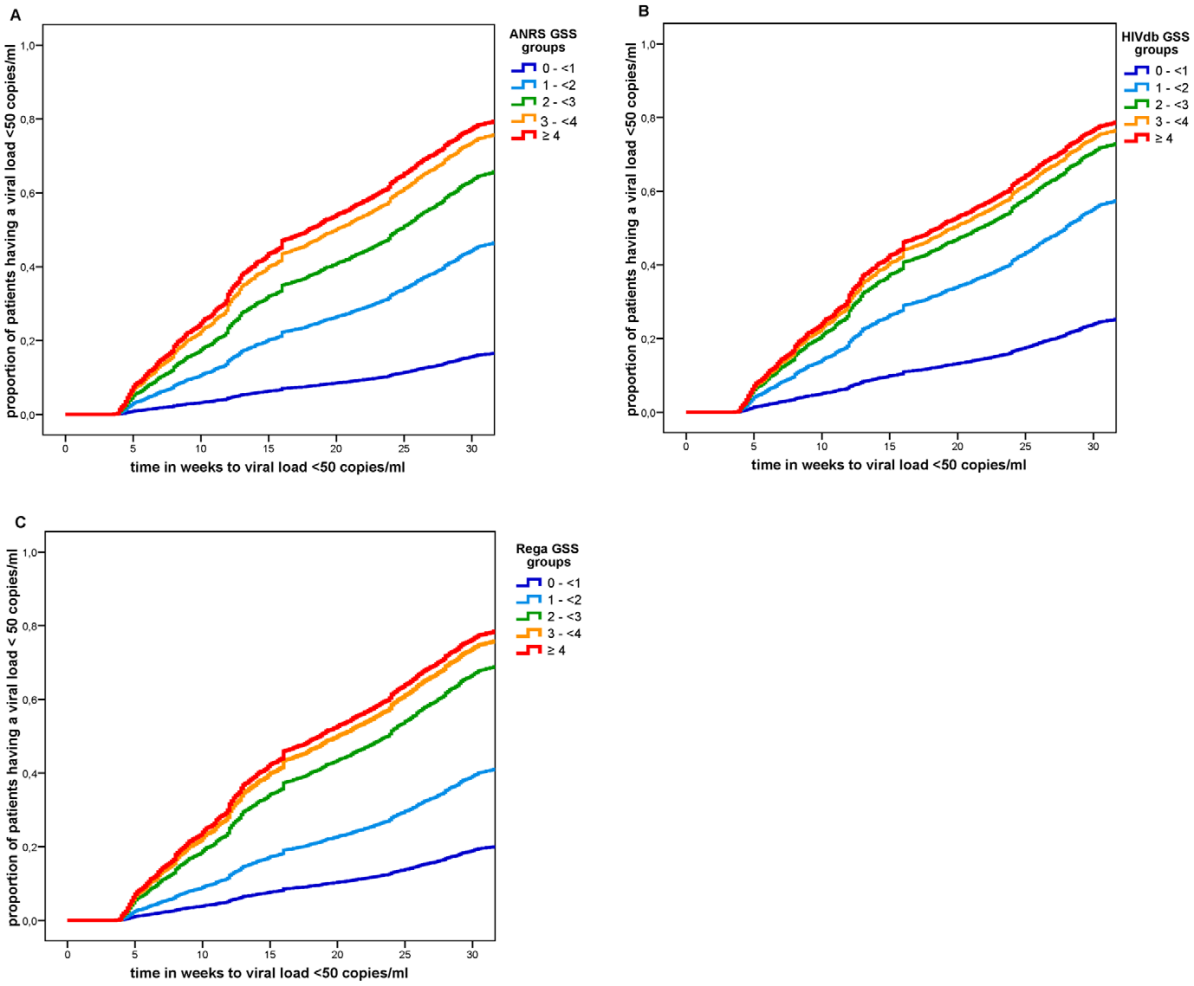
Association of undetectable viral load and Genotypic Susceptibility Score over time. Kaplan Meier curves showing the association between time to undetectable viral load and the proportion of TCEs having an undetectable viral load for the 5 Genotypic Susceptibility Score groups for (A) ANRS (B) HIVdb and (C) Rega. Due to lost to follow-up at later viral load measurement time points, we limited the follow-up time to 30 weeks.

In figure 6, Kaplan-Meier curves are given, showing clear associations between the GSS groups and the proportion of TCEs having an undetectable viral load. The GSS group of 4 or higher show the highest proportion of TCEs having an undetectable viral load. The Odds Ratios of each GSS group are given in Table 4 for all time point measurements. In the comparison between the different GSS groups and the GSS group of 0 to <1, increasing Odds Ratios were found for an increasing GSS. Odds Ratios were higher at week 24 compared to week 12 for all GSS groups and in all three interpretation systems, whereas the results at week 48 did not differ much from those at week 24. Due to the low numbers of included TCEs in GSS group ≥4 and at week 48, large confidence intervals were seen in these groups. At week 24, the Odds Ratios increased from 4.70 (95% CI: 2.57–8.60) to 26.42 (95% CI: 13.49–51.77) for ANRS, from 3.62 (95% CI: 2.56–5.13) to 13.49 (95% CI: 8.25–22.06) for HIVdb, and from 3.46 (95% CI: 2.03–5.91) to 19.34 (10.70–34.94) for Rega.

**Table 4:** Logistic regression for calculating association between undetectable viral load and the GSS groups.

		OR (95% CI)		
system	GSS group	week 12	week 24	week 48
ANRS	0-<1	ref.	ref.	ref.
	1-<2	3.86 (2.07−7.20)	4.70 (2.57−8.60)	5.70 (2.17−14.95)
	2-<3	7.20 (3.94−13,18)	9.89 (5.48−17.83)	11.94 (4.62−30.83)
	3-<4	13.54 (7.50−24.44)	21.24 (11.87−37.99)	23.11 (9.09−58.72)
	≥4	15.01 (7.65−29.45)	26.42 (13.49−51.77)	34.29 (11.56−101.70)
HIVdb	0-<1	ref.	ref.	ref.
	1-<2	2.55 (1.73−3.75)	3.62 (2.56−5.13)	3.19 (1.91−5.33)
	2-<3	5.39 (3.69−7,89)	7.14 (5.06−10.08)	7.21 (4.33−12.00)
	3-<4	8.05 (5.59−11.59)	12.51 (8.9−17.51)	10.06 (6.14−16.46)
	≥4	7.48 (4.51−12.39)	13.49 (8.25−22.06)	12.28 (5.72−26.35)
Rega	0-<1	ref.	ref.	ref.
	1-<2	2.03 (1.20−3.45)	3.46 (2.03−5.91)	2.75 (1.27−5.99)
	2-<3	5.55 (3.41−9.02)	9.72 (5.83−16.22)	8.83 (4.21−18.52)
	3-<4	8.97 (5.61−14.38)	19.15 (11.60−31.61)	15.26 (7.42−31.37)
	≥4	9.73 (5.53−17.11)	19.34 (10.70−34.94)	19.40 (7.93−47.48)

Logistic regression analysis evaluating the association between undetectable viral load and the GSS groups (with GSS group 0-<1 as reference) at different time points for the three interpretation systems. The number of treatment-change episodes for the GSS group 0-<1, 1-<2, 2-<3, 3-<4, and ≥4 are:

178, 389, 485, 959, and 142 at 12 weeks; 157, 433, 638, 1206, and 142 at 24 weeks; 62, 182, 242, 540, and 59 at 48 weeks. These numbers were similar for the three systems.

## Discussion

In this study, data from treated HIV-1 patients were modeled to predict virological outcome comparing genotypic drug resistance with the most commonly used interpretation systems. We used logistic regression and AUC calculations and showed in 3,763 treatment change episodes that ANRS, HIVdb, and Rega, do not differ in predicting virological outcomes.

Comparisons of interpretation systems have been previously reported [9], [10], [15], [16], [17]. In this work, due to the large study population, we were able to compare genotypic susceptibility scores between patients using many different drug therapy combinations and control for important possible confounders. The results of our study were in agreement with previous findings [10], [16]. In addition to previous work, our study has extensively looked at the differences between the prediction ability of the systems at different time points. We both included short term responses (week 12) and longer term responses (week 24 and 48).

An explanation for the findings in this study is that the systems all make use of the same literature available on correlations between genotypic and phenotypic analyses as well as correlations with treatment history and clinical response.

Several studies showed small changes in genotypic susceptibility scores between different systems. For example Ravela et al. [18], that compared 4 different interpretation systems (including ANRS, HIVdb, and Rega), reported a 4.4% complete discordance, with at least 1 system assigning susceptible and another system assigning resistant; 29.2% displayed partially discordance; and 66.4% were complete concordant. However, in this study we found that these differences do not have a large influence on the virological outcome of treatment.

A possible limitation of studies comparing different interpretation systems lies in the translation of the indications from the interpretation systems into numeric values, which are taken arbitrarily. However, we have used the same principles used by authors of HIV drug-resistance algorithms for calculating the genotypic susceptibility score. Therefore we were able to compare the three systems in the way they are used in practice. We also used the Rega scores without the suggestions about weighting of scores for boosted PI drugs and NNRTI. Using these unadjusted scores did not change in GSS distributions and virological outcome to a great extent.

Some novel drugs (etravirine, darunavir, tipranavir) were not frequently used in our study population. Similarly, drugs belonging to the newly approved classes, such as raltegravir and maraviroc, were not included. Therefore, the predictive value we found is not a validation for all individual rules in the system and we did not attempt to validate individual rules. Continuous validations in large dataset with recent drug data will therefore remain needed.

No restriction on therapies was performed; therefore suboptimal regimens (fewer than three full-dose drugs) were included. However, the group of patients receiving suboptimal regimens was small and the same for all three interpretation systems. Furthermore, it was previously demonstrated that removal of suboptimal treatment reduces the accuracy of the models [19].

Much discussion has been going on about which follow-up period is most suitable to validate a system. Short term responses might be more directly attributable to the antiviral drug activity whereas longer term outcomes might be more clinically relevant but more easily confounded by other issues such as loss in adherence, drug discontinuations and switches [20]. In our study less than 1/3 of all cases were left at the 48 week time point measurement. This loss to follow up creates selection bias in this group. Therefore, this 48-week-group may not be representative of the whole study population. The patients, who remain on therapy until the 48^th^ week after start of therapy, will do better on therapy and will have better virological responses than patients who switch to another therapy at earlier stages. In accordance, we found stronger associations between interpretation systems and virological outcomes at later time points compared to earlier time points in the logistic regression analyses. However, in the logistic regression that compared the different GSS groups to the GSS group of 0 to <1, the Odds Ratios were similar between week 24 and week 48. Therefore, week 24 may be a well suitable time point to measure long term responses. However, confidence intervals in week 48 were large, because of low numbers of included TCEs, therefore creating a bias at this time-point.

In conclusion, we found that the three most common used interpretation systems do not differ in their ability to predict virological response. Also, when looking into different time points, the prediction abilities between the systems were similar. Since the overall performance is comparable, these systems might evolve towards a more consistent scoring in the future. New breakthroughs might be needed for further improvement in genotypic resistance test interpretation.

## References

[1] Lorenzi P, Opravil M, Hirschel B, Chave JP, Furrer HJ (1999). Impact of drug resistance mutations on virologic response to salvage therapy. Swiss HIV Cohort Study.. AIDS.

[2] Durant J, Clevenbergh P, Halfon P, Delgiudice P, Porsin S (1999). Drug-resistance genotyping in HIV-1 therapy: the VIRADAPT randomised controlled trial.. Lancet.

[3] Cingolani A, Antinori A, Rizzo MG, Murri R, Ammassari A (2002). Usefulness of monitoring HIV drug resistance and adherence in individuals failing highly active antiretroviral therapy: a randomized study (ARGENTA).. Aids.

[4] Tural C, Ruiz L, Holtzer C, Schapiro J, Viciana P (2002). Clinical utility of HIV-1 genotyping and expert advice: the Havana trial.. Aids.

[5] Meynard JL, Vray M, Morand-Joubert L, Race E, Descamps D (2002). Phenotypic or genotypic resistance testing for choosing antiretroviral therapy after treatment failure: a randomized trial.. Aids.

[6] Shafer RW, Rhee SY, Pillay D, Miller V, Sandstrom P (2007). HIV-1 protease and reverse transcriptase mutations for drug resistance surveillance.. AIDS.

[7] Liu TF, Shafer RW (2006). Web resources for HIV type 1 genotypic-resistance test interpretation.. Clin Infect Dis.

[8] Van Laethem K, De Luca A, Antinori A, Cingolani A, Perna CF (2002). A genotypic drug resistance interpretation algorithm that significantly predicts therapy response in HIV-1-infected patients.. Antivir Ther.

[9] De Luca A, Cingolani A, Di Giambenedetto S, Trotta MP, Baldini F (2003). Variable prediction of antiretroviral treatment outcome by different systems for interpreting genotypic human immunodeficiency virus type 1 drug resistance.. J Infect Dis.

[10] Prosperi MC, Altmann A, Rosen-Zvi M, Aharoni E, Borgulya G (2009). Investigation of expert rule bases, logistic regression, and non-linear machine learning techniques for predicting response to antiretroviral treatment.. Antivir Ther.

[11] Sloot P, Coveney P, Bubak MT, Vandamme AM, B ON (2008). Multi-science decision support for HIV drug resistance treatment.. Stud Health Technol Inform.

[12] Sloot PM, Coveney PV, Ertaylan G, Muller V, Boucher CA (2009). HIV decision support: from molecule to man.. Philos Transact A Math Phys Eng Sci.

[13] Johnson VA, Brun-Vezinet F, Clotet B, Gunthard HF, Kuritzkes DR (2008). Update of the Drug Resistance Mutations in HIV-1.. Top HIV Med.

[14] Hastie T, Tibshirani H, Friedman J (2001). The elements of statistical learning.. Springer.

[15] Zazzi M, Prosperi M, Vicenti I, Di Giambenedetto S, Callegaro A (2009). Rules-based HIV-1 genotypic resistance interpretation systems predict 8 week and 24 week virological antiretroviral treatment outcome and benefit from drug potency weighting.. J Antimicrob Chemother.

[16] Rhee SY, Fessel WJ, Liu TF, Marlowe NM, Rowland CM (2009). Predictive Value of HIV-1 Genotypic Resistance Test Interpretation Algorithms.. J Infect Dis.

[17] Fox ZV, Geretti AM, Kjaer J, Dragsted UB, Phillips AN (2007). The ability of four genotypic interpretation systems to predict virological response to ritonavir-boosted protease inhibitors.. Aids.

[18] Ravela J, Betts BJ, Brun-Vezinet F, Vandamme AM, Descamps D (2003). HIV-1 protease and reverse transcriptase mutation patterns responsible for discordances between genotypic drug resistance interpretation algorithms.. J Acquir Immune Defic Syndr.

[19] Larder B, Wang D, Revell A, Montaner J, Harrigan R (2007). The development of artificial neural networks to predict virological response to combination HIV therapy.. Antivir Ther.

[20] Brun-Vezinet F, Costagliola D, Khaled MA, Calvez V, Clavel F (2004). Clinically validated genotype analysis: guiding principles and statistical concerns.. Antivir Ther.

